# Method for Developing Communication Skills in Autism – DHACA: appearance and content validation

**DOI:** 10.1590/2317-1782/20232023138en

**Published:** 2023-12-18

**Authors:** Ana Cristina de Albuquerque Montenegro, Amanda Gabrielly de Santana Silva, Bianca Queiroga, Rafaella Asfora Lima, Ivana Arrais de Lavor Navarro Xavier

**Affiliations:** 1 Departamento de Fonoaudiologia, Universidade Federal de Pernambuco – UFPE - Recife (PE), Brasil.; 2 Departamento de Psicologia, Inclusão e Educação, Universidade Federal de Pernambuco – UFPE - Recife (PE), Brasil.

**Keywords:** Autism, Communication, Speech, Language and Hearing Sciences, Assistive Technology, Alternative and Augmentative Communication Systems

## Abstract

**Purpose:**

To validate the appearance and content of the DHACA method to develop communication skills in autism.

**Methods:**

This qualitative and quantitative validation study included 10 speech-language-hearing judges with expertise in alternative communication. The judges received the communication book, the description of the principles, skills, and strategies in the DHACA method, and a form with items for them to appraise the appearance and content of the method. The validity was calculated with the content validity index.

**Results:**

The response analysis made it possible to calculate the degree of agreement between judges and develop the new instrument version. The calculation of the content validity index revealed excellent content validity. The judges made suggestions regarding the content of the communication book, texts regarding the participation of communication partners and modeling, using cues, and communicative skills.

**Conclusion:**

The degree of agreement between judges ensured the validation of the appearance and content of the DHACA method, considering the items alone and the whole instrument. Hence, its use can be recommended for speech-language-hearing clinical practice.

## INTRODUCTION

Autism spectrum disorder (ASD) is a neurodevelopmental disorder characterized by changes and impairments in communication and social interaction and restricted and repetitive behavior patterns, interests, and activities, with a wide range of degrees of intensity^(1)^.

Individuals with ASD have heterogeneous linguistic skills, ranging from the absence of speech or production of few words to the acquisition of more robust verbal skills, though with persistent deficits in situations of functional use, aiming at communication^(2)^.

They commonly have delayed language acquisition and development, with linguistic impairments in pragmatic, semantic, morphosyntactic, and phonological aspects. They have limited communicative social functions and usually communicate to request or refuse something. The most used communicative functions are related to meeting their needs or protesting^(2)^.

ASD descriptions also include impairments in shared attention, eye contact, and communicative intention, which influence communication acquisition and development. The more severe the deficit in these skills, the later their communication develops^(2)^.

The neurotypical language acquisition process evokes various biological and social-pragmatic aspects that enable the development of linguistic skills. Hence, it requires the involvement of sociocognitive skills such as comprehension, shared intentionality, and participation in social-communicative activities with linguistically and symbolically competent people^(3)^.

Brazilian studies on the effective use of augmentative and alternative communication (AAC) to develop communication in individuals with ASD are still scarce^(4)^. Most intervention methods used in the country are directly taken from or inspired by protocols developed abroad, which may make them culturally unfeasible or inaccessible to communication partners^(4)^.

Another gap is the viability of intervention in less structured contexts, as alternative communication programs are usually applied in rather structured settings in which interlocutors do not use verbal cues. Hence, it may be unfeasible to generalize responses to real communication situations^(5)^.

The DHACA method (a Portuguese acronym that stands for Development of Communication Skills in Autism)^6^ is grounded on the social-pragmatic theory or theory of usage-based language acquisition^(3)^. The author understands that people acquire language through linguistic activities while interacting with others.

The prerequisites to apply the method as a speech-language-hearing intervention include minimum shared attention, good fine motor coordination, normal eye-hand coordination, and the lack of comorbidities such as intellectual or visual disability. Moreover, imitation skills and symbolic play facilitate method implementation. It must be highlighted that this DHACA version is not indicated to children who do not have the above prerequisites, although it may be adapted in future studies.

This method has six guiding principles and aims to stimulate five skills. The following are the guiding principles: 1) Using visual cues; 2) Shared attention; 3) Participation of communication partners and Modeling; 4) Using cues; 5) Linguistic development; and 6) Functional communication. The skills are as follows: 1) Initial communicative intention; 2) Requesting with fringe vocabulary lexical expansion; 3) Requesting with lexical and morphosyntactic expansion; 4) Morphosyntactic, lexical, and communicative function expansion; and 5) Dialog.

Given the above, DHACA aims to develop functional communication by using a robust alternative communication system as an alternative to bridge the gap in the national literature, as previously mentioned. Therefore, this study aimed to validate the appearance and content of the DHACA method focusing on its recommendation for use in speech-language-hearing clinical practice.

METHOD

## This qualitative-quantitative validation study aimed to validate the appearance and content of the DHACA method to recognize it as reliable and valid to fulfill its purpose. The study was approved by the Human Research Ethics Committee, no. 5.831.912.

Ten speech-language-hearing judges were invited for content validation. They were divided equally into two groups: a) professionals with more than 5 years of clinical experience in speech-language-hearing intervention in individuals with autism and who had been using the DHACA method for more than 12 months; and b) professionals with more than 5 years of clinical experience in speech-language-hearing intervention in individuals with autism but who were not using the DHACA method.

After they had been selected, each judge received an invitation letter via e-mail to participate in the research, along with an informed consent form and a form for the validation of the appearance and content of the DHACA method.

DHACA uses a communication book with 66 pictograms on a single page and smaller overlapping tabs with other vocabulary pictograms, organized according to lexical categories, and gradually included in the therapeutic process^(6)^. These overlapping tabs, organized according to lexical categories, have a single line with 10 pictograms, above which the corresponding words are written. The vocabulary in the method was selected based on the concept of “core words” and “fringe words”. Core words are those more frequently used, mainly pronouns, verbs, adjectives and adverbs, which occur often in general interactions. Fringe words are less often used, mainly nouns that tend to be specific to the AAC user’s context and interest^(7)^.

The organization of the core words vocabulary in the DHACA communication book characterizes it as a robust alternative communication system^(8)^.

The initial version of the method resulted from literature analysis and clinical practice of the speech-language-hearing researchers who developed the method. It was submitted to the judges to analyze the content with the following procedures:

- Presenting the communication book (Appendix A) and describing the principles and skills of the DHACA method and their respective strategies.
- Analyzing the appearance and content of the communication book by filling out a content analysis form, with which the researchers calculated the content validation index (CVI)^(9)^ and appraised qualitative aspects pointed out by the examiners.

The answer options in the form sent to the judges were as follows: 1 = Not representative; 2 = Little representative; 3 = Representative item; and 4 = Very representative. Besides the score, there was room for possible suggestions, which generated the study's qualitative data.

Each item’s relevance score (I-CVI) was calculated by adding the agreement in items checked with “3” or “4” by specialists and dividing it by the total number of answers^(9)^. The total instrument’s relevance score (T-CVI) was calculated by dividing the total number of items considered relevant by the judges by the total number of items^(9)^.

Changing or excluding techniques, strategies, and appearance in the book was based on the agreement of at least two judges per item.

After the changes, the material was again presented for the judges’ appraisal, which did not lead to any further changes.

## RESULTS

The study will first present data on the specialists’ responses regarding the appearance of the book – which includes its physical form and visual aspects. In both the 1^st^ and 2^nd^ rounds, five judges considered it representative, and the other five considered it very representative.

The interrater agreement rate per item regarding the content of the communication book and principles and skills approached in the method reached values above 78% - i.e., above the minimum to be considered representative – as early as the initial analysis. The summarized analysis of the frequency of judges’ responses is shown in Table 1.

**Table 1:** Distribution of judges according to responses on method representativity (N = 10)

**1^st^ Round**				
**Variable**	**Not representative**	**Little representative**	**Representative item**	**Very representative**
Communication book appearance	-	-	5	5
Communication book content	-	-	5	5
PRINCIPLES				
Communication book	-	-	2	8
Using visual aid	-	-	2	8
Promoting shared attention	-	-	1	9
Participation of communication partner and modeling	-	2	4	4
Using cues	-	-	2	8
Linguistic development	-	-	2	8
Functional communication	-	1	2	7
SKILLS				
Request with single pictogram	-	1	2	7
Request with fringe vocabulary	-	1	2	7
Request with expanded fringe vocabulary	-	-	2	8
Morphosyntactic and pragmatic expansion	-	-	4	6
Narrative	-	1	1	8
**2^nd^ Round**				
**Variable**	**Not representative**	**Little representative**	**Representative item**	**Very representative**
Communication book appearance	-	-	5	5
Communication book content	-	-	2	8
PRINCIPLES				
Using visual aid	-	-	-	10
Shared attention	-	-	1	9
Participation of communication partner and modeling	-	-	2	8
Using cues	-	1	4	6
Linguistic development	-	-	-	10
Functional communication	-	-	1	9
SKILLS				
Initial communicative intention	-	-	1	9
Request with fringe vocabulary lexical expansion	-	-	-	10
Request with lexical and morphosyntactic expansion	-	-	2	8
Morphosyntactic, lexical, and communicative function expansion	-	-	-	10
Dialog	-	-	-	10

Likewise, the whole instrument analysis reached 99% of agreement, surpassing the minimum rate to consider it representative (90%). In other words, the instrument was validated even in the first analysis, as the I-CVI and T-CVI agreement rates were above the minimum percentage of 0.78 to be considered representative (Table 2).

**Table 2:** Content Validation Index of individual items and the whole instrument

**1st Round**														
				**Principles**						**Skills**				
		CBA	CBC	UVA	PSA	PCPM	UC	LD	FC	RSP	RFV	REFV	MPE	NT
I-CVI		1	1	1	1	0.8	1	0.9	0.9	1	1	1	1	1
T-CVI: 0.96 - 96%														
**2^nd^ Round**														
				**Principles**						**Skills**				
		CBA	CBC	UVA	SA	PCPM	UC	LD	FC	ICI	RFVLE	REFV	MLCFE	DL
I-CVI		1	1	1	1	1	0.9	1	1	1	1	1	1	1
T-CVI : 0.99 - 99%														

Caption: CBA = communication book appearance; CBC = communication book content; UVA = using visual aids; SA = shared attention; PSA = promoting shared attention; PCPM = participation of the communication partner and modeling; UC = using cues; LD = linguistic development; FC = functional communication; RSP = request with single pictograms; RFV = request with fringe vocabulary; REFV = request with expanded fringe vocabulary; MPE = morphosyntactic and pragmatic expansion; NT = narrative; ICI = initial communicative intention; RFVLE = request with fringe vocabulary lexical expansion; MLCFE = morphosyntactic, lexical, and communicative function expansion; DL = dialog; CVI = Content Validation Index; I-CVI = analyzes each item separately; T-CVI = analyzes the whole instrument

It is important to point out that specialists made suggestions for some items that scored “3 = representative item” or “4 = very representative” to improve the method. However, only the items in which at least two judges agreed were changed or excluded from the text.

The excerpt below presents the judges’ main suggestions

1. DHACA book, concerning appearance and content

“Colored backgrounds in the categories would be more interesting than just the borders”. “I miss pictograms for ‘my turn’ and ‘your turn’”. “I have never used the demonstrative pronouns ‘this’ and ‘that’, so I suggest reviewing these pictograms”.

1. Principle: Participation of communication partners and modeling

“The principle is ‘Participation of the communication partner and modeling’, but the description focuses on modeling – whereas the partner is responsible for modeling... I think it is important to define ‘partner’ (an important role in AAC development)”.

1. Principle: Using cues

“I do not always see the need for total physical support to teach a new skill. I prefer the hierarchy of cues from less to greater (verbal, visual, physical). This makes the intervention less invasive and more based on a naturalistic profile”.

1. Principle: Using visual cues

“About the term ‘visual cues’, would it not be interesting to add, ‘participating in an activity together with therapists and another one or two people with complex communication needs to show the use and reach of AAC? In such a strategy, other pairs could illustrate this powerful communication tool”.

1. Principle: Functional communication

“I suggest including the promotion of communicative functions other than comments and information”. “I suggest including an item ‘Dialog’ – creating a conversation moment: How are you today? Is it going to be sunny or rainy?... etc. Everything that is clearly described gains room”.

Based on the suggestions, some changes were made to the appearance and content of the DHACA book and the guiding principles. In appearance, the background of the core vocabulary pictograms was colored according to their parts of speech, instead of having colors only on the borders. As for book content, the demonstrative pronouns “this” and “that” were removed, while “my” and “your” were included”.

The researcher made the appropriate changes in the protocol, based on the specialists’ analyses in the first round. The revised instrument (second version) was again sent to the specialist judges to assess the material, now changed, continuing the appearance and content validation process.

In the second assessment round, the final version of the instrument reached a high level of agreement between evaluators concerning its appearance and content.

The quantitative analysis of the judges’ responses in the reassessment demonstrated that no further changes were needed in the content of the method, thanks to the I-CVI values above 0.78 in all items and 0.92 T-CVI. Reassessment responses, following the reformulation, were more homogeneous, which ensured greater reliability, as seen in Table 2. Furthermore, the few suggestions made in the reassessment were not considered because they addressed issues related to lexical category personalization criteria, which are organized according to the person’s needs and demands, as described in the principle “linguistic development”.

Since no further changes were needed because of the I-CVI values above 0.78 in all method items, the appearance and content were maintained as defined in this stage. Hence, the DHACA method was considered validated regarding its appearance and content, as it reached a 0.99 T-CVI, fulfilling its purpose.

Thus, the final description of the principles of the DHACA method, with their goals and strategies, is shown in Chart 1, and that of the communicational skills, with their goals and strategies, is shown in Chart 2.

**Chart 1:** Description of principles in the DHACA method, with objectives and strategies (final version).

**Principle**	**Description**
**Using visual aid**	Individuals with Autism spectrum disorder (ASD) are generally considered “visual thinkers” or concrete thinkers, as they have greater skills in visual memory tasks than in hearing memory ones – which, in turn, require greater abstraction capacity^(10)^. Visual resources include pictograms, which are linguistic symbols – i.e., meaning is conveyed through a picture that represents a concept/meaning, just as the spoken word conveys a meaning. This representation is social in nature – i.e., it is taught in the linguistic community to which the person belongs.
	Hence, when children have interactive access to symbols in augmentative and alternative communication (AAC) resources, they internalize their meaning and develop communication, using the symbols whose meanings fit the communication with a partner. Thus, the function of AAC with pictograms is potentially identical to the function of words. Therefore, AAC has been used early to help the acquisition and development of verbal language and social interaction in children with good prognoses of verbal language development and help structure nonverbal language and expression in nonverbal children^(11)^.
**Shared attention**	Shared attention is a greatly relevant skill in the social-pragmatic theory^(3)^. It occurs in situations of social interaction between three parties, in which the child and the child share experiences regarding a third object/event/symbol used as a reference. It is an essential skill for language development because it helps the person understand other people as interaction agents and the objective of communication. Through shared attention, children learn to use a communication symbol with adults just as they used it with them^(12)^.
**Participation of the communication partner and modeling**	Communication partners are those who partake in communicative interactions with someone who communicates using AAC. They must be able to understand the child’s way of communicating, including speech, nonverbal communication, and any ACC method they use.
	Communication partners who effectively use AAC are essential interaction partners for children to learn communication using their alternative communication system. People with complex communication needs require greater engagement and specific strategies on the part of their interlocutors to fully participate in communicative activities^(13)^. Therefore, it is essential to train their communication partners, especially those at home, as they are the social participants who have the greatest, deepest, and most powerful influence on these people’s language development and/or recovery. Moreover, they must be apt to adequately model alternative communication and respond to the child with the same set of communication types or modes^(14)^.
	Modeling in AAC is a strategy used by communication partners when they intend to be the interlocutor model for the AAC user to learn to use their AAC system – first by imitating them and then using it independently. Thus, modeling using the robust AAC system ensures greater morphosyntactic, semantic, and pragmatic comprehension as it allows the child and partners to use the communication system, in natural contexts and a variety of communication situations^(15)^.
	Modeling can occur in three ways: first, by directly talking with the child – e.g., when the communication partner is speaking and points to or somehow calls attention to vocabulary items in the alternative communication system. The second is when they want the child to imitate them immediately, if possible. So, the communication partner shows how to use it, as if they were the child speaking, responding, commenting, for the child to imitate the partner. Lastly, the third way of modeling is by showing other people using the alternative communication system – e.g., showing a video of other children using an alternative communication book^(16)^.
	The authors of a systematic review^(15)^ state that modeling ensures significant linguistic gains in a) pragmatics, by increasing communication turns; b) semantics, by increasing receptive and expressive vocabulary; c) syntax, by increasing sentences with more words; and d) morphology, by increasing the use of morphological variations.
	Every communication partner must be apt to use the communication book. Thus, speech-language-hearing therapists responsible for implementing the DHACA method must train relatives, other health and education professionals who attend to these children, and their social partners to use the DHACA communication book.
	Children who often use AAC need communication partners who can support them to integrate them into everyday activities, such as their school routine. These children’s successful communication requires their inclusion in settings where people can use AAC. This helps them construct better communication spaces and encourages the use of indicated resources.
**Using cues**	Using concrete cues, such as visual and tactile ones, increases the efficiency of teaching new skills to children with ASD, ensuring greater comprehension and motivation to do tasks, considering these children’s ease of interaction with visual stimuli^(10)^.
	Using physical cues when beginning to teach skills is a strategy used with children with ASD due to their difficulty ideating movements. Thus, when they learn to point with visual or verbal cues, physical cues are removed.
	According to Berger^(17)^, people with ASD have asymmetrical sensory information processing. Hence, the author justifies using sensory support, including touch, amplified proprioception, and multimodal sensory input (audios combined with visual models to support independent movements) to aid psychomotor development.
	Time delay is another strategy, in which the therapist uses fewer prompts when instructing. For instance, the interval between initial instruction and any additional instruction or stimulus increases gradually as the person becomes more proficient in the skill they are being taught^(17)^.
	It is important to highlight that the cues are gradually removed as the child gains communication autonomy.
**Linguistic development**	The method promotes functional communication development by following typical morphosyntactic, lexical, and pragmatic development. Pragmatic skills are developed along with the following communication functions:
	**Initial functions**
	- Communicative intention: using the language to satisfy an immediate desire. Asking for objects, animals, help, actions, people, etc.; responding to others.
	- Protesting: disagreeing with or repressing someone else’s action, interrupting their speech or action with a desired action.
	- Naming: spontaneously naming objects, people, and actions.
	**Intermediate functions**
	- Informative/interrogative: asking questions with interrogative pronouns (who, when, which, where, etc.).
	- Commenting: making comments, giving spontaneous information, showing something, expressing pain, giving opinions, and sharing ideas.
	- Expressing feelings: expressing gratitude and feelings.
	- Social interaction: greeting, saying goodbye, thanking, apologizing, and showing off.
	**Advanced functions**
	- Reporting: telling a fact or retelling a story.
	- Imagination: creating a story or telling a joke.
	- Conversation: maintaining a conversation.
	The main DHACA tool is an AAC book in A4 paper size, with pictograms organized in a grid – A3 paper size can also be used. The vocabulary represented by the pictograms was selected based on the concept of “core words” and “fringe words”^(7)^. Core words comprise precisely the core ones in the language, mostly verbs, adjectives, adverbs, and pronouns, and rarely nouns, which are very often used in different social contexts. Presenting them as core vocabulary helps the person use various communication functions, such as asking questions, asking for things, refusing, protesting, commenting, and describing. The core vocabulary generally remains in the same place in the communication book to minimize the demands on their memory and motor planning.
	Altogether 66 pictograms comprise the grid on a single page (Appendix A - Figure 1A). These words, focused on linguistic development, were chosen based on the core word survey described in Franco’s thesis^(18)^. The core vocabulary book page is not personalized. Fringe words comprise nouns and a range of words more related to specific contexts of interest of the AAC user^(15)^. They are in smaller overlapping pages, forming a single-line grid with 10 pictograms, organized by lexical category (Appendix A - Figure 2A). Some categories may have two or more pages. Personalized lexical categories are gradually included during the therapeutic process, and their vocabulary is selected based on the following criteria: user’s preference, such as foods, toys, YouTube channels, etc.; core and fringe word survey^(18)^, such as qualities, actions, etc.; lexical development, such as colors, numbers, alphabet, geometric shapes, animals, means of transportation, people, places, parts of the body, etc.; pragmatic development: something to say, feelings, etc.; specific needs according to social contexts, such as personal hygiene, school supplies, etc.
**Functional communication**	The final goal of this method is to promote functional communication in people with ASD – i.e., the ability to receive and convey messages effectively and independently, according to the requirements of the social context, favoring the development of communication skills. This broad definition refers to the whole process, rather than isolated aspects such as speech intelligibility, comprehension, reading, and so forth. It includes all verbal and nonverbal communication modalities and focuses on communicative efficiency and independence, such as appropriate responses to everyday circumstances^(19)^.
	Implementing AAC use by individuals with ASD in social interaction requires the planning of interactive situations that promote dialog exchanges between the subject and their interlocutors. Caregivers, health professionals, and classmates must be instructed to stimulate communication with the child in all social settings.

**Chart 2:** Description of skills in the DHACA method, with objectives and strategies (final version).

**Skill**	**Objectives**	**Strategies**
**Initial communicative intention**	The child should have communicative intention, requesting something from the interlocutor, pointing to the pictograms I + WANT (in the communication book) + single pictogram^*^ of what they desire. The child should build the sentence in sequence, pointing to the respective pictograms, possibly accompanied by speech. To advance to the next skill, the child must be able to discriminate up to four single pictograms and independently (i.e., spontaneously, without cues) build the sentence I + WANT + pictogram.	1. The therapist initially places a single pictogram related to an item of the child’s preference at the top of the core vocabulary page.
		2. The activity should be prepared to trigger in the child the desire to ask for preferred items.
		3. As the child shows interest in objects, the therapist uses physical and verbal cues to help them point to the pictograms that form the sentence: I + WANT + item. Visual cues are used to gradually remove physical ones. If the child has difficulty identifying I + WANT, colored adhesive tape can be used to indicate the pictograms.
		4. When the child is able to perform the above task, another pictogram is included; physical cues may be used again.
		5. The therapist uses the communication book to guide their talk with the child, pointing to pictograms in the core vocabulary page, modeling their communication – i.e., pointing to the pictograms of the words they are saying.
		6. Enable parents to use the book continuously, showing how to use it in the therapeutic setting, and train other relatives and professionals from various contexts.
		7. When the child uses up to four pictograms to build the sentence, discriminating each one by independently pointing at them, they can move on the develop the next skill.
		8. The therapist must instruct caregivers to promote skill acquisition, by showing how they use it with the child and then ask the caregiver to practice with the child in the therapeutic setting. The therapist must help the caregiver with feedback during practice.
**Requesting with lexical expansion from the fringe vocabulary**	The child should be able to ask the interlocutor for something by pointing to the pictograms I + WANT + a pictogram in the fringe vocabulary from one of the two tabs with distinct lexical categories. They should build the sentence in sequence by pointing to the pictograms, possibly accompanied by speech. To advance to the next skill, the child must request with the construction “I + WANT + a pictogram in the fringe vocabulary”, independently, spontaneously, and without cues.	1. The therapist should dismiss the single pictograms and bind with a spiral initially one or two tabs of fringe vocabulary pictograms. Each tab has a line with up to 10 pictograms in lexical categories related to the items of preference used in the previous skill.
		2. The activity must be planned to trigger in the child the desire to ask for some of their preferred items.
		3. When the child wants an object but cannot ask for it by pointing independently at it, they must be guided with physical, visual, and/or verbal cues or modeling to point to the pictograms of the sentence: I + WANT + item in one of the fringe vocabulary tabs.
		4. The therapist begins using the communication book by talking to the child, pointing to pictograms in the core vocabulary page and fringe vocabulary tabs, modeling their communication (i.e., pointing to the pictograms of the words they are speaking), and enabling parents to use the book continuously, as demonstrated in the therapeutic setting. They should also train other relatives and professionals from different contexts.
		5. The therapist can model by showing the child how to form the desired sentences.
		6. When the child is able to ask for up to two different items in the fringe vocabulary, pointing at them independently, they can move on to develop the next skill.
		7. The therapist must instruct caregivers to help the child acquire the skill, demonstrating how to do it with the child and then having the caregiver practice with the child in the therapeutic setting. Thus, they enable parents to use it continuously and train other relatives and professionals from various contexts. The therapist must help caregivers, by providing feedback during practice.
**Requesting with lexical and morphosyntactic expansion**	The child should be able to form sentences with the pictograms: I + WANT + two pictograms from either the fringe or core vocabulary. Three or more tabs should be added. They should build the sentence in sequence by pointing to the pictograms, possibly accompanied by speech. To advance to the next skill, the child must be able to point to I + WANT + two pictograms from either the fringe or core vocabulary, independently, spontaneously, without cues.	1. The therapist must include fringe vocabulary tabs such as foods, toys, qualities, and places, besides the ones used in the previous skill.
		2. The activity must be planned to trigger in the child the desire to ask for preferred items.
		3. When the child desires an object, action, person, or some help but has difficulty finding the desired pictogram or leafing through the tabs, they can be helped with physical, visual, and/or verbal cues or modeling to leaf through them independently and point at the pictograms of the sentence: I + WANT + two pictograms from either the fringe or core vocabulary.
		4. The therapist must use the communication book by speaking to the child, pointing to pictograms in the core vocabulary page and fringe vocabulary tabs, and modeling their communication.
		5. The therapist can model by demonstrating to the child how to form the desired sentences.
		6. When the child is able to ask for up to two items from different tabs, leaf through the tabs independently, and point to the pictograms of the sentence I + WANT + two pictograms from either the fringe or core vocabulary independently, they can move on to develop the next skill.
		7. The therapist must instruct caregivers to help acquire the skill by demonstrating how to use the book with the child and then asking them to practice with the child in the therapeutic setting. The therapist must help the caregiver with feedback during practice, enabling them to use it continuously and train other relatives and professionals from various contexts.
		8. The family must be encouraged to further use new words from the core and fringe vocabulary stimulated during the sessions.
**Morphosyntactic, lexical, and communicative function expansion**	The child should be able to form sentences with three or more words, with different goals:	1. The therapist can include new fringe vocabulary tabs, such as feelings, the notion of time, other verbs, and social greetings, besides the tabs used in the previous skill.
	Developing communicative functions:	2. The activity should be planned to encourage the development of communicative functions with four or more pictograms.
	● Informative/interrogative function, asking questions with interrogative pronouns (who, when, which, where, etc.).	3. When the child wants to use any communicative function and has difficulties finding the desired pictogram or leafing through the tabs, they can be helped with physical, visual, and/or verbal cues or modeling to point at the four pictograms independently.
	● Commenting: making comments, giving spontaneous information, showing something, demonstrating pain, giving opinions, and sharing ideas.	4. The therapist must use the communication book to speak to the child, pointing at pictograms in the core vocabulary page or fringe vocabulary tabs, and modeling their communication.
	● Expressing feelings, gratitude.	5. The therapist can model by demonstrating to the child how to form the desired sentences.
	● Social-interactive function: greeting, saying goodbye, thanking, apologizing, and showing off.	6. The therapist can use structured visual resources to help develop the skill.
	They should build the sentence in sequence by pointing to the pictograms, possibly accompanied by speech. They must use them with interlocutors in various contexts to begin acquiring the next skill.	7. When the child is able to form sentences with four or more words with different pragmatic goals, using items from different tabs, and leafing through the tabs independently, then they can move on to develop the next skill.
		8. The therapist must instruct the caregiver to help the child acquire the skill by demonstrating how to use the book with the child and then having them practice with the child in the therapeutic setting. The therapist must help caregivers with feedback during practice, enabling them to use it continuously, and training other relatives and professionals from various contexts.
		9. The family must be encouraged to further use new words from the core and fringe vocabulary used to stimulate the child to form new sentences during the sessions.
**Dialog**	The child should be able to use the following communicative functions:	1. The therapist can insert new fringe vocabulary tabs, according to the child’s and family’s demands in the child’s various social and school contexts.
	Reporting: telling a fact or retelling a story.	2. The activity must be free to encourage dialog using various communicative functions, maintaining conversation, creating, telling, and retelling stories, and reporting facts.
	Imagination: creating a story or telling a joke.	3. When the child wants to use any communicative function but has difficulties finding the desired pictogram, they can be helped by rephrasing with modeling.
	Conversation: maintaining a conversation.	4. The therapist must use the communication book to speak to the child, modeling their communication.
	They should build the sentence in sequence by pointing to the pictograms, possibly accompanied by speech. They must use it with interlocutors in various contexts.	5. The therapist can also model by demonstrating to the child how to form the desired sentences.
		6. The therapist can use structured visual resources to help the child develop the skill.
		8. The therapist must instruct caregivers to help the child acquire the skill by demonstrating how to use the book with the child and then including the caregiver in the conversation for them to also use the AAC book with the child and the therapist. The therapist must help the caregiver with feedback during practice enabling them to use it continuously and train other relatives and professionals from various contexts.
		9. Acquiring this skill indicates independence to use the DHACA communication book, beginning the assisted discharge process, and allowing caregivers to insert new tabs as needed.

* Single pictogram – loose laminated pictogram, separate from the fixed communication board

## DISCUSSION

The DHACA method was developed based on the scientific knowledge and clinical experience of the speech-language-hearing researchers who developed it throughout their professional history working with AAC in children with autism.

This method uses a robust communication system aiming to bridge the gaps in AAC effectiveness in communication development, the feasibility of implementing it in less structured and more naturalistic contexts, and the possibility of having it clinically reproduced in speech-language-hearing intervention in this population^(5)^.

The process of validating an intervention method, particularly an AAC one in Brazil, is an innovative factor due to the absence of methods developed for the national context.

The difference in this method is that it is based on the social-pragmatic theory and the linguistic development theory, emphasizing the role of communication partners and the use of various natural contexts. It is a Brazilian method whose linguistic basis is the functional use of the language, from selecting the vocabulary to using communicative functions. Moreover, it considers cultural and individual specificities.

This pioneering Brazilian intervention method describes the skills to be gradually acquired by people with ASD based on linguistic development throughout the intervention, finally aiming at the acquisition of functional communication. It uses a robust, easy-to-handle communication system – a communication book whose core vocabulary comprises pictograms selected based on a bank with high-frequency words^(18)^ and fringe vocabulary.

After the analysis of the judges’ agreement in the first assessment, the CVI result validated the appearance and content of the DHACA method^(9)^. However, relevant qualitative suggestions were made, leading to adjustments that potentialized the appearance and content of the method. It must be emphasized that five judges are professionals with clinical experience in using other AAC intervention methods. Hence, their approval reinforces the validation of the method.

The suggestions included coloring the background of the pictograms in the core vocabulary according to their parts of speech, instead of having only the borders colored. This change reinforces the concept of a semantic color-coding system, which was already used in the method, but only in the borders. Thus, concepts are grouped regarding their grammatical role, facilitating their use and memorization, and helping children develop their grammar.

Another change was the removal of the demonstrative pronouns “this” and “that” and the inclusion of “my” and “your”. Studies indicate that the acquisition and use of personal and possessive pronouns are related to the interlocutor’s skill in recognizing themselves in their relationship with others and understanding their and the other person’s perspective – i.e., the development of social communication skills^(20)^. Besides these possessive pronouns being quite frequently used as well, this resource stimulates the use of first-person pronouns among people with ASD, who have difficulties in this aspect^(21)^.

Selecting core words is crucial to obtain good results. Both the selection and organization of vocabulary are essential tasks for successful AAC use. It must provide access to a large vocabulary that is adequate for communication development and organized to make it easier for children to retrieve pictograms^(18)^.

The suggestion about the content on the participation of communication partners led to highlighting their importance in the skill development process and the use of the resource in various contexts and settings, which agrees with the purpose of the method with a social-pragmatic approach^(6)^.

It is believed that the initial use of physical cues favors psychomotor development and the acquisition of new skills. Such cues include sensory support with touches, amplified proprioception, and multimodal sensory input (audios combined with visual models to aid independent movements)^(22)^.

Judges also suggested including different communicative functions in the “Functional communication” text. Hence, it included the description of communicative functions in “Linguistic development”, classifying them as initial, intermediate, and advanced, and the terminology of the DHACA skills was changed. Robust communication systems allow for exploring and developing various communicative functions along with morphosyntactic, semantic, and pragmatic progress^(8)^. Furthermore, an adequate selection of core and fringe vocabulary enables children to express themselves according to these various communicative functions^(18)^.

As for the last skill, its name was changed from narrative to dialog, considering that its goal is to have functional communication – which involves dialog, rather than only narrative communication.

The gaps in scientifically described AAC intervention methods highlight the importance of validating the content and appearance of the DHACA method. Moreover, evidence-based practices in decision-making are essential to increase the quality of therapeutic intervention.

The DHACA method makes way for further AAC intervention studies in people with ASD, helping improve clinical practices regarding language disorders associated with ASD. As for future perspectives, further studies are being planned to advance in other validation stages with other psychometric properties.

## CONCLUSION

The study validated the content and appearance of the DHACA method, fulfilling the validation stages defined in the literature.

This innovative method contributes to Brazilian speech-language-hearing therapy, considering the importance of using validated instruments and evidence-based practices.

## Figures and Tables

**Figure 1A.**
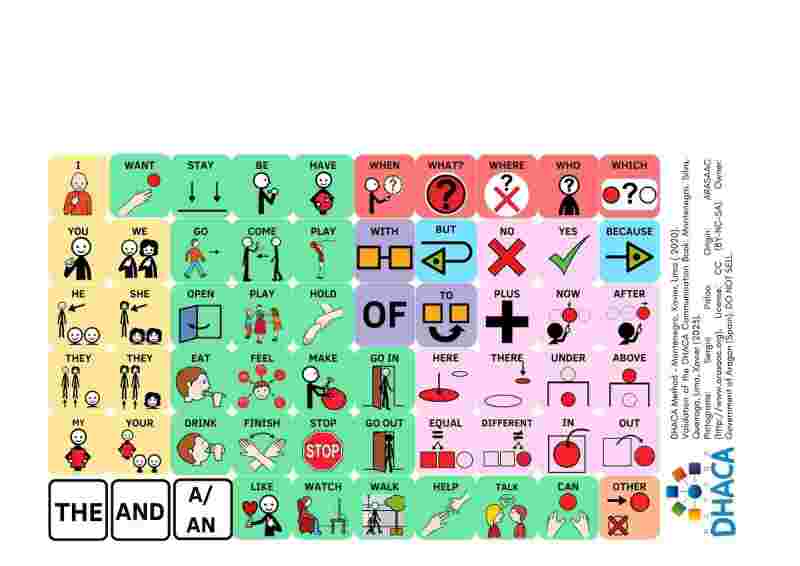
Essential vocabulary page in DHACA Method

**Figure 2A.**
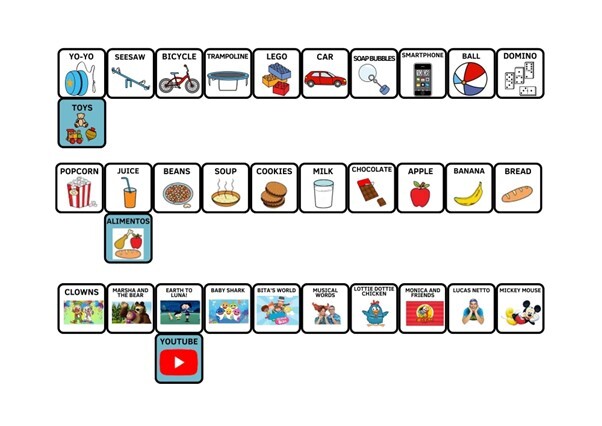

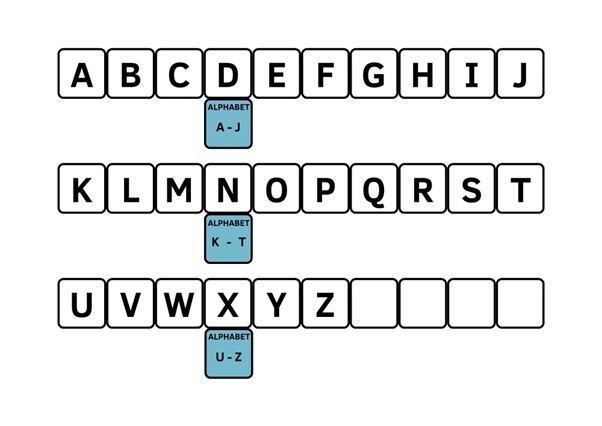

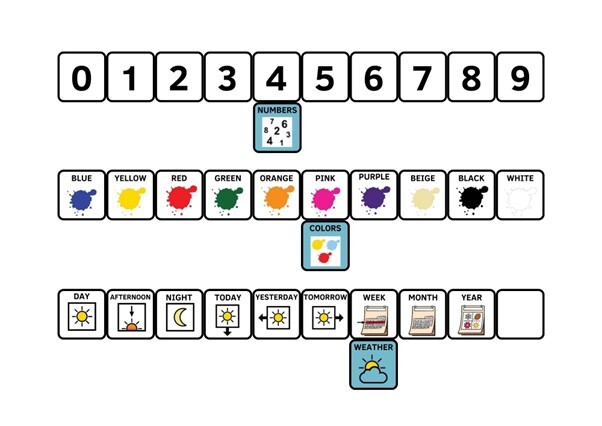

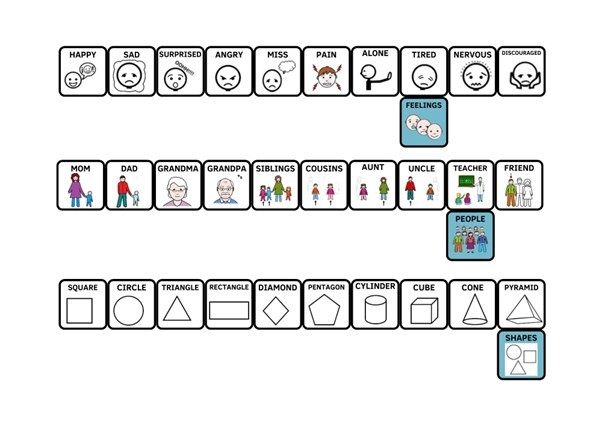

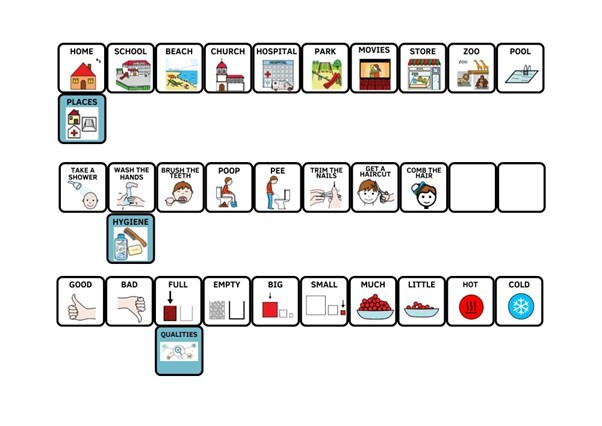

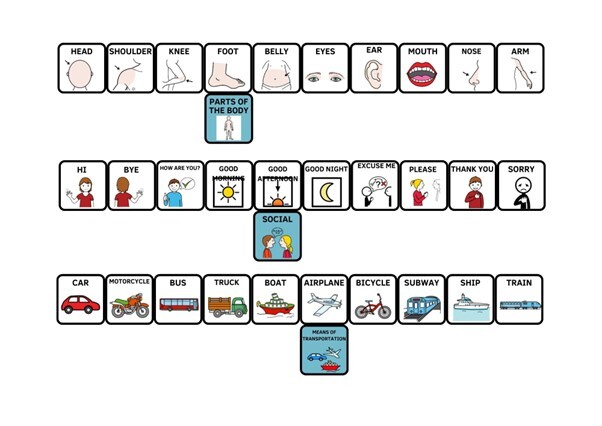

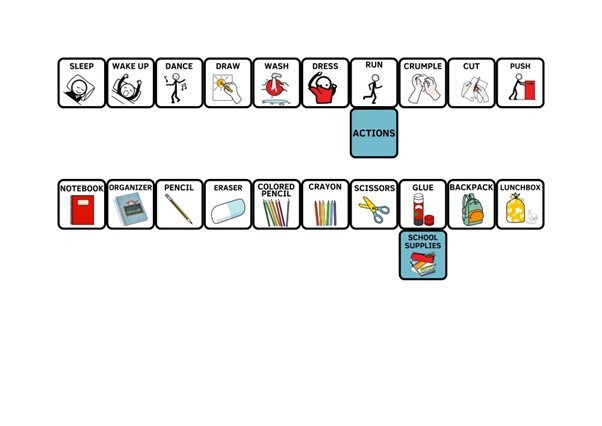
Examples of secondary vocabulary tabs in the DHACA method
