# Supplementary material for: Method for Developing Communication Skills in Autism – DHACA: appearance and content validation
Source: Codas. 2023 Dec 18;36(3):e20230138. doi: 10.1590/2317-1782/20232023138en (PMC10750986; doi:10.1590/2317-1782/20232023138en)
Supplement: Translation

# Translation: Método de Desenvolvimento das Habilidades de Comunicação no Autismo – *DHACA*: validação da aparência e do conteúdo

**DOI:** 10.1590/2317-1782/20232023138pt

**Authors:** Ana Cristina de Albuquerque Montenegro, Amanda Gabrielly de Santana Silva, Bianca Queiroga, Rafaella Asfora Lima, Ivana Arrais de Lavor Navarro Xavier

**Affiliations:** 1 Departamento de Fonoaudiologia, Universidade Federal de Pernambuco – UFPE - Recife (PE), Brasil.; 2 Departamento de Psicologia, Inclusão e Educação, Universidade Federal de Pernambuco – UFPE - Recife (PE), Brasil.

**Keywords:** Autismo, Comunicação, Fonoaudiologia, Tecnologia Assistiva, Sistemas de Comunicação Alternativos e Aumentativos

## Abstract

**Objetivo:**

Validar a aparência e o conteúdo do método de Desenvolvimento das Habilidades de Comunicação no Autismo (DHACA).

**Método:**

Trata-se de estudo de validação de abordagem quali-quantitativa. Participaram do estudo dez juízes fonoaudiólogos com expertise na área da comunicação alternativa. Os juízes receberam o livro de comunicação, bem como a descrição dos princípios, habilidades e estratégias do método DHACA e um formulário com itens relativos à apreciação da aparência e conteúdo do método. A validade foi calculada usando o índice de validade de conteúdo.

**Resultados:**

A análise das respostas possibilitou o cálculo do grau de concordância entre os juízes e a elaboração da nova versão do instrumento. O cálculo do Índice de Validade de Conteúdo revelou uma validade de conteúdo excelente. Os juízes deram sugestões referentes aos aspectos de conteúdo do livro de comunicação, nos textos de participação do parceiro de comunicação e modelagem, uso de dicas e habilidades comunicativas.

**Conclusão:**

O grau de concordância observado entre os juízes possibilitou a obtenção da validação da aparência e do conteúdo do método DHACA, considerando-se os itens isoladamente e o instrumento como um todo, podendo ter seu uso recomendado na prática clínica fonoaudiológica.

## INTRODUÇÃO

O Transtorno do Espectro do Autismo (TEA) é um transtorno do neurodesenvolvimento que se caracteriza por alterações e prejuízos na comunicação e na interação social, bem como em padrões restritos e repetitivos de comportamentos, interesses e atividades, apresentando grande variação no grau de intensidade^(1)^.

As habilidades linguísticas nos indivíduos com TEA são heterogêneas, variando entre a ausência de fala ou produção de poucas palavras, a indivíduos que adquirem habilidades verbais mais robustas, entretanto com déficits persistentes em situações de uso funcional, visando a comunicação^(2)^.

São comuns atrasos na aquisição e desenvolvimento da linguagem, com comprometimentos linguísticos nos aspectos pragmático, semântico, morfossintático e fonológico. As funções sociais da comunicação são limitadas, geralmente o indivíduo se comunica para solicitar ou rejeitar algo, sendo as funções comunicativas mais frequentemente usadas aquelas relacionadas à satisfação de necessidades e ao protesto^(2)^.

No TEA também são descritos comprometimentos na Atenção Compartilhada (AC), contato visual e intenção comunicativa, habilidades que influenciam a aquisição e desenvolvimento da comunicação. Quanto mais grave o déficit nestas habilidades, mais tardio é o desenvolvimento da comunicação^(2)^.

Durante o processo de aquisição da linguagem neurotípica são evocados vários aspectos biológicos e sociopragmáticos que tornam possível o desenvolvimento de habilidades linguísticas, sendo assim, é necessário o envolvimento de habilidades sociocognitivas como a compreensão, o compartilhamento da intencionalidade e a participação em atividades sociocomunicativas com indivíduos humanos linguístico e simbolicamente competentes^(3)^.

No Brasil, ainda são escassos os estudos quanto à eficácia do uso da Comunicação Alternativa Aumentativa (CAA) no desenvolvimento da comunicação de indivíduos com TEA^(4)^. A maioria dos métodos de intervenção utilizados no país são diretamente tomados ou inspirados em protocolos desenvolvidos no exterior, o que pode torná-los culturalmente inviáveis ou inacessíveis para os parceiros de comunicação^(4)^.

Outro hiato encontrado é viabilidade da intervenção em contextos menos estruturados, uma vez que os programas alternativos de comunicação são, usualmente, aplicados em ambientes extremamente estruturados e sem uso dicas verbais por parte do interlocutor, podendo inviabilizar a generalização das respostas para situações reais de comunicação^(5)^.

O método DHACA - Desenvolvimento das Habilidades de Comunicação no Autismo^(6)^, tem como pressuposto a Teoria Sociopragmática ou “Teoria da aquisição da linguagem baseada no uso”^(3)^. O autor entende que é através da atividade linguística, durante a interação com o outro, que o ser humano adquire sua linguagem.

O método, proposto como intervenção fonoaudiológica, possui como pré-requisitos para a aplicação: presença mínima de atenção compartilhada, boa coordenação motora fina, ausência de alterações na coordenação óculo-manual, ausência de comorbidades como deficiência intelectual, deficiência visual. Destaca-se que a presença da habilidade de imitação e do brincar simbólico facilitam a implementação do método. É importante ressaltar que esta versão do DHACA não é indicada para quem não apresenta os pré-requisitos acima descritos, entretanto, poderá ser adaptada em estudos futuros.

Além disso, o método possui seis princípios norteadores e cinco habilidades a serem estimuladas. Os princípios norteadores são: 1) Uso de Apoio Visual; 2) Atenção Compartilhada; 3) Participação do parceiro de comunicação e Modelagem; 4) Uso de dicas; 5) Desenvolvimento Linguístico e 6) Comunicação Funcional. As habilidades são: 1) Intenção comunicativa inicial; 2) Pedido com ampliação lexical no vocabulário acessório; 3) Pedido com ampliação lexical e morfossintática; 4) Ampliação morfossintática, lexical e das funções comunicativas e 5) Diálogo.

Com base no exposto, o DHACA tem como objetivo desenvolver a comunicação funcional por meio do uso de um sistema robusto de comunicação alternativa, buscando ser uma alternativa diante das lacunas observadas na literatura nacional, como apontado anteriormente. Assim, o objetivo do presente estudo foi validar a aparência e o conteúdo do método DHACA, com foco na recomendação do seu uso na prática clínica fonoaudiológica.

## MÉTODO

Trata-se de um estudo de validação, com abordagem quali-quantitativa, que visa validar a aparência e conteúdo do Método DHACA, de modo a reconhecê-lo como confiável e válido para o fim a que se destina e foi aprovado pelo Comitê de Ética em Pesquisa com Seres Humanos, n° 5.831.912.

Para a validação de conteúdo, foram selecionados dez juízes, fonoaudiólogos, divididos, igualmente, em dois grupos: a) com experiência clínica em intervenção fonoaudiológica para indivíduos com autismo há mais de cinco anos e que estejam aplicando o método DHACA há mais de doze meses e b) com experiência clínica em intervenção fonoaudiológica para indivíduos com autismo há mais de cinco anos, mas que não apliquem o método DHACA.

Após seleção, foi encaminhada, para cada juiz, uma carta convite por e-mail, convidando-os a participarem da pesquisa, bem como o Termo de Consentimento livre e Esclarecido (TCLE), acompanhado do formulário para a validação da aparência e do conteúdo do método DHACA.

O método DHACA utiliza um livro de comunicação, composto por sessenta e seis pictogramas numa única página, além de páginas menores sobrepostas, com outros pictogramas de vocabulários, que são divididos em categorias lexicais, inseridos paulatinamente durante o processo terapêutico^(6)^. Essas páginas sobrepostas têm apenas uma linha composta por dez pictogramas com a escrita dos nomes acima e são separadas de acordo com a categoria lexical. A seleção do vocabulário do método foi baseada no conceito de “*core words*” e “*fringe words*”. *Core words* (palavras essenciais) consiste em palavras de alta frequência, contendo, principalmente, pronomes, verbos, adjetivos e advérbios. Essas palavras são muito frequentes nas interações de modo geral. *Fringe words* (palavras acessórias) consiste em palavras de baixa frequência, principalmente substantivos, que tendem a ser específicos do contexto e de interesse do usuário de CAA^(7)^.

A organização do vocabulário no livro de comunicação DHACA tendo como base o conceito de *core words*, caracteriza-se como um sistema robusto de comunicação alternativa^(8)^.

A versão inicial do método, resultante da análise da literatura e da prática clínica das fonoaudiólogas pesquisadoras que desenvolveram o método, foi submetida aos juízes para a realização da análise de conteúdo, por meio dos seguintes procedimentos:

- Apresentação do livro de comunicação (Apêndice A), bem como a descrição dos princípios e habilidades do método DHACA e suas respectivas estratégias;
- Análise da aparência e do conteúdo do livro de comunicação, por meio do preenchimento de um formulário de análise de conteúdo, que possibilitou o cálculo do Índice de Validade de Conteúdo (IVC)^(9)^, bem como a apreciação de aspectos qualitativos apontados pelos examinadores.

No formulário que foi enviado aos juízes constavam como opções de respostas as seguintes possibilidades: 1= Não representativo; 2= Pouco representativo; 3= Item representativo e 4= Bastante representativo. Além do espaço para pontuação, tinha um espaço para possíveis sugestões, que geraram os dados qualitativos deste estudo.

O escore do índice de relevância dos itens individualmente (IVC - I) foi calculado por meio da soma de concordância dos itens que foram marcados por “3” ou “4” pelos especialistas e divididos pelo número total de respostas^(9)^. Já para calcular o escore do índice de relevância do instrumento como um todo (IVC - T), foi realizada a divisão do número total de itens considerados como relevantes pelos juízes pelo número total de itens^(9)^.

É importante salientar que para a realização de mudanças ou exclusão de técnicas, estratégias e aparência do livro foi considerada a concordância entre, pelo menos, dois juízes, em cada item.

Na sequência, após as modificações, o material foi novamente apresentado para apreciação dos juízes, o que não resultou em novas mudanças.

## RESULTADOS

Inicialmente serão apresentados os dados relativos às respostas dos especialistas no que diz respeito à aparência do livro - que inclui a forma física e o aspecto visual do instrumento. Na 1ª e na 2ª rodadas observou-se que, em ambas, cinco juízes consideraram-na representativa e cinco bastante representativa.

Quanto ao conteúdo do livro de comunicação e aos princípios e habilidades do método, a taxa de concordância entre os juízes para os itens analisados, individualmente, mostraram valores acima de 78%, ou seja, do mínimo para ser considerado representativo, já na análise inicial. O compilado da análise da frequência das respostas dos juízes também pode ser observado na Tabela 1.

**Tabela 1:** Distribuição de juízes segundo respostas sobre a representatividade do método (N=10)

**1ª Rodada**				
**Variável**	**Não representativo**	**Pouco representativo**	**Item representativo**	**Bastante representativo**
Aparência do livro de comunicação	-	-	5	5
Conteúdo do livro de comunicação	-	-	5	5
PRINCÍPIOS				
Livro de comunicação	-	-	2	8
Uso de apoio visual	-	-	2	8
Promoção da Atenção Compartilhada	-	-	1	9
Participação do parceiro de comunicação e Modelagem	-	2	4	4
Uso de dicas	-	-	2	8
Desenvolvimento Linguístico	-	-	2	8
Comunicação Funcional	-	1	2	7
HABILIDADES				
Pedido com pictograma avulso	-	1	2	7
Pedido com vocabulário acessório	-	1	2	7
Pedido com ampliação de vocabulários acessórios	-	-	2	8
Ampliação morfossintática e pragmática	-	-	4	6
Narrativa	-	1	1	8
**2ª Rodada**				
**Variável**	**Não representativo**	**Pouco representativo**	**Item representativo**	**Bastante representativo**
Aparência do livro de comunicação	-	-	5	5
Conteúdo do livro de comunicação	-	-	2	8
PRINCÍPIOS				
Uso de Apoio Visual	-	-	-	10
Atenção Compartilhada	-	-	1	9
Participação do parceiro de comunicação e Modelagem	-	-	2	8
Uso de dicas	-	1	4	6
Desenvolvimento Linguístico	-	-	-	10
Comunicação Funcional	-	-	1	9
HABILIDADES				
Intenção comunicativa inicial	-	-	1	9
Pedido com ampliação lexical no vocabulário acessório	-	-	-	10
Pedido com ampliação lexical e morfossintática	-	-	2	8
Ampliação morfossintática, lexical e das funções comunicativas	-	-	-	10
Diálogo	-	-	-	10

Da mesma forma, o resultado da análise do instrumento como um todo, que foi de 99% de concordância, superou a taxa mínima para torná-lo representativo (90%). Ou seja, ainda na primeira análise, o instrumento foi validado, uma vez que a taxa de concordância do IVC - I e do IVC - T foram acima do valor percentual mínimo de 0,78, valor mínimo para ser considerado representativo (Tabela 2).

**Tabela 2:** Índice de Validação de Conteúdo dos itens individuais, bem como do instrumento como um todo

**1ª Rodada**														
				**Princípios**						**Habilidades**				
		ALC	CLC	UAV	PAC	PPCM	UD	DL	CF	PPA	PVA	PAVA	AMP	NT
IVC - I		1	1	1	1	0,8	1	0,9	0,9	1	1	1	1	1
IVC - T: 0,96 - 96%														
**2ª Rodada**														
				**Princípios**						**Habilidades**				
		ALC	CLC	UAV	AC	PPCM	UD	DL	CF	ICI	PALVA	PAVA	AMLFC	DL
IVC - I		1	1	1	1	1	0,9	1	1	1	1	1	1	1
IVC - T: 0,99 - 99%														

Legenda: ALC = aparência do livro de comunicação; CLC = conteúdo do livro de comunicação; UAV = uso de apoio visual; PAC = promoção da atenção compartilhada; AC = atenção compartilhada; PPCM = participação do parceiro de comunicação e modelagem; UD = uso de dicas; DL = desenvolvimento linguístico; CF = Comunicação Funcional; PPA = pedido com pictograma avulso; PVA = pedido com vocabulário acessório; PAVA = pedido com ampliação de vocabulários acessório; AMP = ampliação morfossintática e pragmática; NT = narrativa; ICI = intenção comunicativa inicial; PALVA = pedido com ampliação lexical no vocabulário acessório; AMLFC = ampliação morfossintática, lexical e das funções comunicativas; DL = diálogo; IVC = Índice de Validade de Conteúdo; IVC-I = Permite analisar cada item individualmente; IVC-T = Permite analisar o instrumento como um todo

Vale ressaltar que alguns itens julgados como “3= item representativo” ou “4= bastante representativo” também receberam sugestões por parte dos especialistas, visando ao aprimoramento do método. Todavia, apenas os itens que apresentaram concordância, entre pelo menos dois juízes, foram modificados ou excluídos do texto.

O recorte abaixo descreve as principais sugestões dadas pelos juízes:

1. Livro DHACA, quanto a aparência e conteúdo

“O fundo todo na cor da categoria seria mais interessante do que só a borda.” “Sinto falta dos pictogramas “minha vez e sua vez.” “Até o momento não utilizei os pronomes demonstrativos “isto ” e “aquilo”, por esse motivo sugiro a revisão desses pictogramas.”

1. Princípio: Participação do parceiro de comunicação e modelagem

“O princípio é Participação do Parceiro de Comunicação e Modelagem, a descrição está focando a modelagem - e o parceiro se torna o responsável por realizar a modelagem... acho importante definir o parceiro (figura importante no desenvolvimento da CAA).”

1. Princípio: Uso de dicas

“Nem sempre vejo a necessidade de suporte físico total para ensinar nova habilidade. Prefiro hierarquia de dica de menos para mais (verbal, visual, física). Dessa forma a intervenção fica menos invasiva e mais pautada em perfil naturalístico.”

1. Princípio: Uso de apoio visual

“Sobre o termo “apoio visual”, será que não seria interessante acrescentar: participar de uma atividade conjunta com terapeutas e mais uma ou duas pessoas com necessidades complexas de comunicação para mostrar o uso e o alcance da Comunicação Suplementar e Alternativa (CSA), seria uma estratégia onde outras díades poderão ilustrar essa poderosa ferramenta de comunicação.”

1. Princípio: Comunicação funcional

“Eu incluiria promover as diferentes funções comunicativas, não só comentários e informações.” “Colocaria o item ‘diálogo’ - fazer com que haja um momento de conversa: como você está hoje? Será que teremos sol ou chuva?... etc. Tudo que fica claramente descrito começa a ter espaço.”

Diante das sugestões foram realizadas modificações relacionadas à aparência e conteúdo do livro DHACA e aos princípios norteadores. Quanto à aparência, aplicou-se preenchimento do fundo dos pictogramas do vocabulário essencial, na cor da classe gramatical correspondente, em vez de apenas a borda colorida. No que se refere ao conteúdo do livro houve a retirada dos pronomes demonstrativos “isto” e “aquilo”, e inclusão de “meu/minha”, “seu/sua.”

Os pesquisadores procederam às mudanças no protocolo, realizando as modificações pertinentes, de acordo com as análises dos especialistas na primeira rodada. O instrumento revisado, em sua 2ª versão, foi novamente enviado aos juízes especialistas para que avaliassem o material, agora modificado, dando seguimento à validação da aparência e do conteúdo.

Na segunda rodada de avaliação, a versão final do instrumento obteve um alto nível de concordância entre os avaliadores, quanto à aparência e ao conteúdo.

Após a análise quantitativa das respostas dos juízes na reavaliação, constatou-se que não houve necessidade de modificações no conteúdo do método, devido aos valores de IVC – I acima de 0,78 para todos os itens e IVC – T de 0,92. Destaca-se que, após a reformulação, as respostas recebidas na reavaliação foram mais homogêneas, trazendo mais confiabilidade, como pode ser observado na Tabela 2. Além disso, as poucas sugestões que foram dadas na reavaliação não foram consideradas, porque referiam-se a questões relacionadas ao critério de personalização das categorias lexicais, que, conforme a descrição do princípio “desenvolvimento linguístico”, são organizadas de acordo com as necessidades e demandas do indivíduo.

Como não houve necessidade de modificações, devido aos valores de IVC - I acima de 0,78 para todos os itens do método, foi mantida a aparência e conteúdo desta etapa, podendo-se considerar o método DHACA validado em sua aparência e em seu conteúdo, já que obteve IVC – T de 0,99, atendendo à sua finalidade.

Sendo assim, segue a apresentação final da descrição dos princípios do método DHACA, com objetivos e estratégias no Quadro 1 e das habilidades comunicacionais com objetivos e estratégias, no Quadro 2.

**Quadro 1:** Descrição dos princípios do método DHACA, com objetivos e estratégias, versão final

**Princípio**	**Descrição**
**Uso de Apoio Visual**	Os indivíduos com Transtorno do Espectro do Autismo (TEA), em geral, são considerados “pensadores visuais” ou pensadores concretos, pois apresentam uma maior habilidade em tarefas de memória visual em detrimento de atividades de memória auditiva que, por sua vez, demandam maior capacidade de abstração^(10)^. Dentre os recursos visuais, têm-se os pictogramas que são símbolos linguísticos, isto é, o significante é um desenho que representa um conceito/significado, da mesma forma que a palavra falada é um significante que representa um significado. Esta representação é de caráter social, isto é, é ensinada dentro da comunidade linguística em que o indivíduo está inserido.
	Desse modo, quando as crianças têm acesso aos símbolos de um recurso de Comunicação Alternativa Aumentativa (CAA), em situações de interação, elas irão internalizar, significar e desenvolver a sua comunicação, utilizando os símbolos que terão seus significados de acordo com o uso junto aos parceiros de comunicação. Assim, a função da CAA, com o uso dos pictogramas, é potencialmente idêntica à função da palavra. Diante do exposto, a CAA vem sendo utilizada precocemente, no intuito de promover a aquisição e o desenvolvimento da linguagem verbal e da interação social, em crianças com bom prognóstico de desenvolvimento de linguagem verbal; e de contribuir para a estruturação de linguagem e expressão não verbal, nos casos de crianças não verbais^(11)^.
**Atenção Compartilhada**	A Atenção Compartilhada (AC) é uma habilidade com grande relevância dentro da Teoria Sociopragmática^(3)^. A AC ocorre em situações de interação social triádica em que a criança e o adulto dividem experiências em relação a um terceiro objeto/evento/símbolo referente. É uma habilidade imprescindível para o desenvolvimento da linguagem, porque propicia ao indivíduo a compreensão do outro como agente intencional, entendendo, dessa forma, o objetivo do ato comunicativo. Através da AC, a criança aprende a utilizar um símbolo comunicativo dirigido ao adulto da mesma forma como este o fez em relação a ela^(12)^.
**Participação do Parceiro de Comunicação e Modelagem**	O parceiro de comunicação é aquele envolvido em interações comunicativas com um indivíduo que se comunica por meio de um sistema de CAA. Esse deve ser capaz de entender a forma como a criança está se comunicando, incluindo comunicação falada, comunicação não verbal, bem como qualquer modo de CAA usado pela criança.
	Os parceiros de comunicação que usam eficientemente a CAA são parceiros de interação essenciais para que as crianças aprendam a se comunicar usando seu sistema de comunicação alternativa. Pessoas com necessidades complexas de comunicação necessitam de maior engajamento e utilização de estratégias específicas por parte de seus interlocutores para conseguirem atingir ampla participação nas atividades comunicativas^(13)^, portanto é fundamental que seus parceiros de comunicação, especialmente os do meio familiar, sejam capacitados, considerando que são estes atores sociais os que exercem a mais profunda e potente influência no desenvolvimento e/ou recuperação da linguagem dessas pessoas. Outrossim, devem estar aptos a modelar adequadamente a comunicação alternativa e responder à criança por meio desse mesmo conjunto de tipos ou modos de comunicação^(14)^.
	A modelagem no uso da CAA é uma estratégia utilizada pelo parceiro de comunicação quando este tem a intenção de ser modelo de interlocutor para que o usuário de CAA aprenda a utilizar o seu sistema de CAA, inicialmente imitando e, em seguida, utilizando de forma independente. Dessa forma, a modelagem com o uso do sistema robusto de CAA propicia uma melhor compreensão morfossintática, semântica e pragmática por oportunizar o uso do sistema pela criança e pelos parceiros de comunicação, em contextos naturais e situações comunicativas diversas^(15)^.
	A modelagem pode ocorrer de três formas: a primeira é ao conversar diretamente com a criança, por exemplo, enquanto o parceiro de comunicação está falando, aponta ou, de alguma forma, chama a atenção do sujeito para os itens do vocabulário no sistema de comunicação alternativa. A segunda é quando se deseja que a criança imite imediatamente, se possível. Então, o parceiro de comunicação demonstra o uso, como se fosse a criança falando, respondendo, comentando, com o intuito de que ela possa imitá-lo. Finalmente, a terceira forma de modelagem é a partir da demonstração de outras pessoas utilizando o sistema de comunicação alternativa, como por exemplo apresentar um vídeo de outra criança utilizando um livro de comunicação alternativa^(16)^.
	Em uma revisão sistemática^(15)^, os autores afirmam que a modelagem promove ganhos linguísticos significativos na a) pragmática, por aumentos nos turnos de comunicação na b) semântica, marcada pelo aumento do vocabulário receptivo e expressivo, na c) sintaxe, marcada por aumentos de enunciados com mais palavras; e na d) morfologia, marcada por aumentos no uso de variações morfológicas.
	Qualquer parceiro de comunicação deve estar apto a utilizar o livro de comunicação. Deste modo, é imprescindível que o fonoaudiólogo responsável pela implementação do método DHACA capacite familiares, demais profissionais que assistem a criança que utilizará o livro de comunicação com o método DHACA, profissionais da educação, bem como parceiros de círculos sociais diversos da criança.
	A criança que utiliza CAA frequentemente necessita de parceiros de comunicação que possam oferecer suporte para integrá-la nas atividades cotidianas, como por exemplo, sua rotina escolar. Para o sucesso na comunicação destas crianças, é importante que ela esteja inserida em um ambiente no qual as pessoas saibam utilizar a CAA. Isto irá favorecer a construção de melhores espaços comunicativos, além de estimular a prática de uso dos recursos indicados.
**Uso de Dicas**	O uso de dicas concretas, como pistas visuais e táteis, aumenta a eficiência no ensino de novas habilidades para crianças com TEA, garantindo maior compreensão e motivação para realização das atividades propostas, considerando a facilidade que essas crianças apresentam para interagir com os estímulos visuais^(10)^.
	O uso de dica física, no início do ensino das habilidades, é uma estratégia utilizada com crianças com TEA decorrente da dificuldade na ideação de movimentos. Assim que a criança começa a apresentar o apontar com dica visual ou verbal, a dica física é retirada.
	De acordo com Berger^(17)^, pessoas com TEA apresentam uma assimetria de processamento de informações sensoriais. Com isso, a autora justifica o uso de suportes sensoriais incluindo dicas de toque, propriocepção amplificada, entradas sensoriais multimodais (uso de áudio combinado com modelo visual para dar suporte ao movimento independente), para favorecer o desenvolvimento psicomotor.
	Uma outra estratégia utilizada é o atraso de tempo. Isto é uma estratégia que diminui o uso de prompts durante a instrução do terapeuta. Por exemplo, o intervalo de tempo entre a instrução inicial e qualquer instrução ou estímulo adicional aumenta gradualmente à medida que o indivíduo se torna mais proficiente na habilidade que está sendo ensinada^(17)^.
	Vale ressaltar que as dicas são retiradas paulatinamente, à medida que a criança vai apresentando autonomia na sua comunicação.
**Desenvolvimento Linguístico**	A proposta do método em promover o desenvolvimento da comunicação funcional segue o desenvolvimento morfossintático, lexical e pragmático típico. Para o desenvolvimento das habilidades pragmáticas, o método promove o desenvolvimento das funções comunicativas, a seguir:
	**Funções iniciais**
	- Função de intenção comunicativa: uso da linguagem com o objetivo de satisfazer desejo imediato. Solicitação de objetos, animais, ajuda, ações, pessoas etc; responder ao outro;
	- Função de protesto: ir contra ação do outro, reprimir a ação do outro, interromper com fala ou ação uma ação desejada;
	- Função de nomeação: nomeação espontânea de objetos, pessoas e ações.
	**Funções intermediárias**
	- Função de informação/função interrogativa: utilização de perguntas com os pronomes interrogativos (quem, quando, qual, onde etc);
	- Função de comentários: realizar comentários, informações espontâneas, demonstrar algo, demonstrar dor, dar opinião, expressar ideias;
	- Função de expressar sentimentos: expressar gratidão, expressar sentimentos;
	- Função sociointerativa: realizar saudações, despedidas, agradecimentos, pedir desculpas e se exibir;
	**Funções avançadas**
	- Função de relato: contar um fato ou recontar estórias;
	- Função imaginativa: criar uma estória ou contar piadas;
	- Função conversacional: sustentar uma conversa;
	O método DHACA utiliza como ferramenta principal um livro de comunicação aumentativa e alternativa, tamanho A4, com pictogramas organizados em uma grade, que também pode ser confeccionado em tamanho A3. A seleção do vocabulário, representado pelos pictogramas, é baseada no conceito de “core words” e “fringe words”^(7)^. Core words (palavras essenciais) integram uma seleção de palavras composta por palavras nucleares da língua, que são em geral verbos, adjetivos, advérbios e pronomes, e raramente substantivos. Essas palavras são altamente frequentes em diferentes contextos sociais. Ao serem utilizadas como vocabulário essencial permite que o indivíduo faça uso de várias funções comunicativas, como fazer perguntas, solicitar, rejeitar, protestar, comentar e descrever. O vocabulário essencial, de maneira geral, permanece no mesmo local no livro de comunicação, para minimizar as demandas de memória e de planejamento motor.
	Foram selecionados sessenta e seis pictogramas, para compor a grade em uma única página (Apêndice A - Figura 1A). Essas palavras foram selecionadas com foco no desenvolvimento linguístico, baseadas no levantamento de “Core Words”, descritos na tese de Franco^(18)^. Essa página do livro, com o vocabulário essencial, não é personalizada. “Fringe words” (Palavras Acessórias) representam os substantivos e uma gama de palavras mais atreladas a contextos específicos e de interesse do usuário de CAA^(15)^. Elas estão em páginas menores, sobrepostas, em uma grade, de uma única linha, composta por dez pictogramas e são separadas de acordo com a categoria lexical (Apêndice A - Figura 2A). Entretanto, algumas categorias podem ter duas ou mais páginas. As categorias lexicais são inseridas, de modo personalizado, paulatinamente durante o processo terapêutico e o vocabulário das categorias são selecionados de acordo com os seguintes critérios: preferências do usuário, por exemplo: alimentos, brinquedos, vídeos do YouTube etc); levantamento de “Core” e “Fringe Words”^(18)^, por exemplo: atributos, ações; desenvolvimento lexical, por exemplo: cores, números, alfabeto, formas geométricas, animais, meios de transporte, pessoas, lugares, partes do corpo etc; desenvolvimento pragmático: algo a dizer, sentimentos etc; demandas específicas, a partir de contextos sociais, como: higiene pessoal, materiais escolares etc;
**Comunicação Funcional**	O objetivo final do método é promover a comunicação funcional no indivíduo com TEA, destacando que a comunicação funcional é a habilidade de receber e transmitir mensagens, de modo efetivo e independente, de acordo com as exigências do contexto social, favorecendo o desenvolvimento de habilidades comunicativas. Esta definição é abrangente e diz respeito à totalidade do processo e não somente a um aspecto isolado, como inteligibilidade de fala, compreensão, leitura e outros. Inclui toda modalidade de comunicação verbal e não verbal e enfoca a eficiência e independência comunicativa como respostas apropriadas para a demanda diária^(19)^.
	Vale salientar que durante a implementação do uso de CAA pelo indivíduo com TEA em contexto de interação social, demanda o planejamento de situações interativas que promovem as trocas dialógicas entre o sujeito e os interlocutores. Destaca-se que os cuidadores, os profissionais de saúde e escolares devem ser orientados a estimular a comunicação da criança em todos os ambientes sociais.

**Quadro 2:** Descrição das habilidades do método DHACA, com objetivos e estratégias, versão final

**Habilidade**	**Objetivos**	**Estratégias**
**Intenção comunicativa inicial**	A criança deve ser capaz de apresentar intenção comunicativa solicitando algo ao interlocutor, apontando para os pictogramas EU + QUERO (no livro de comunicação) + pictograma avulso^*^ do que deseja. A construção da frase pela criança deve ocorrer de forma sequenciada, apontando para os pictogramas respectivos, podendo ser acompanhado da fala. Para avançar para a próxima habilidade, será necessário que a criança discrimine até 4 pictogramas avulsos e construa a frase Eu+QUERO+pictograma de forma independente, isto é, espontânea, sem dicas.	1. O terapeuta deve colocar, inicialmente, um pictograma solto, referente a um item de preferência da criança, na parte superior da página do vocabulário principal.
		2. A atividade deve ser preparada de modo que promova o desejo da criança em solicitar algum dos itens preferidos.
		3. A criança, ao desejar o objeto de interesse, deve ser direcionada, com dica física, acompanhada de dica verbal, para apontar os pictogramas da frase: EU + QUERO + item. Pode ser utilizada a dica visual, para que a dica física seja retirada paulatinamente. Caso a criança tenha dificuldade em discriminar o EU+QUERO, pode ser utilizada fita adesiva colorida, realçando os pictogramas.
		4. Quando a criança conseguir realizar o quesito acima, deve ser inserido mais um pictograma, podendo voltar a utilizar a dica física.
		5. O terapeuta deve utilizar o livro de comunicação ao direcionar sua fala à criança, apontando pictogramas da página de vocabulário principal, modelando a sua comunicação, isto é, apontando para os pictogramas das palavras que estiver falando.
		6. Empoderar os pais quanto ao uso contínuo, demonstrando o uso no setting terapêutico, bem como capacitar outros familiares e profissionais de contextos diversos.
		7. Quando forem inseridos até quatro pictogramas e a criança realizar a frase, discriminando cada pictograma, apontando de forma independente, pode seguir para promover o desenvolvimento da habilidade seguinte.
		8. O terapeuta deve orientar o cuidador a promover a aquisição da habilidade, devendo ser ensinado por meio de demonstração do terapeuta junto à criança e, em seguida, solicitar que o cuidador pratique junto à criança dentro do setting terapêutico. O terapeuta deve auxiliar o cuidador, dando feedbacks durante a prática.
**Pedido com ampliação lexical no vocabulário acessório**	A criança deve ser capaz de solicitar algo ao interlocutor, apontando para os pictogramas EU + QUERO + um pictograma do vocabulário acessório de uma das duas abas de categorias lexicais distintas. A construção da frase ocorre de forma sequenciada, apontando para os pictogramas, podendo ser acompanhada da fala. Para avançar para a habilidade seguinte, a criança terá que solicitar a partir da construção frasal “EU + QUERO + um pictograma do vocabulário acessório”, de forma independente, espontânea, sem dicas.	1. O terapeuta deve descartar os pictogramas soltos e inserir inicialmente, com espiral, uma ou duas abas de pictogramas de vocabulários acessórios. Cada aba contém uma linha de até 10 pictogramas de categorias lexicais relacionados aos itens de preferência utilizados na habilidade anterior.
		2. A atividade deve ser planejada de modo que promova o desejo da criança em solicitar algum dos itens preferidos.
		3. A criança, ao desejar o objeto, caso não consiga solicitar apontando de forma independente, deve ser direcionada, com dica física, visual e/ou verbal, ou com modelagem, para apontar os pictogramas da frase: EU+ QUERO+ item de uma das abas do vocabulário acessório.
		4. O terapeuta deve iniciar a utilização do livro de comunicação, ao direcionar sua fala à criança, apontando pictogramas da página de vocabulário essencial e do vocabulário acessório, modelando a sua comunicação. Isto é, apontando para os pictogramas das palavras que estiver falando e empoderar os pais quanto ao uso contínuo, demonstrando o uso no setting terapêutico, bem como capacitar outros familiares e profissionais de contextos diversos.
		5. O terapeuta pode modelar, demonstrando à criança a formação de frases que deseja realizar.
		6. Quando a criança conseguir solicitar até dois itens diversos do vocabulário acessório, apontando de forma independente, pode seguir para promover o desenvolvimento da habilidade seguinte.
		7. O terapeuta deve orientar o cuidador a promover a aquisição da habilidade, devendo ser ensinado por meio de demonstração do terapeuta junto à criança e, em seguida, solicitar que o cuidador pratique junto à criança, dentro do setting terapêutico, empoderando os pais quanto ao uso contínuo, bem como capacitar outros familiares e profissionais de contextos diversos. O terapeuta deve auxiliar o cuidador, dando feedbacks durante a prática.
**Pedido com ampliação lexical e morfossintática**	A criança é capaz de formar frases com os pictogramas: EU + QUERO + dois pictogramas (podem ser do vocabulário acessório ou do vocabulário essencial). Devem ser acrescentadas três ou mais abas. A construção da frase deve ocorrer de forma sequenciada, a criança deve apontar para os pictogramas, podendo ser acompanhada da fala. Para avançar para a próxima habilidade, a criança deve apontar para EU + QUERO + dois pictogramas (podem ser do vocabulário acessório ou do vocabulário essencial), de forma independente, espontânea, sem dicas.	1. O terapeuta deve inserir abas do vocabulário acessório como: alimentos, brinquedos, atributos, locais, além das abas utilizadas na habilidade anterior.
		2. A atividade deve ser planejada de modo que promova o desejo da criança em solicitar algum dos itens preferidos.
		3. A criança, ao desejar o objeto, ação, pessoas, ajuda, caso tenha dificuldade em discriminar onde está o pictograma desejado, ou tenha dificuldade em folhear as abas, pode ser auxiliada com dica física, visual e/ou verbal, ou com modelagem, para folhear de forma independente e apontar os pictogramas da frase: EU + QUERO + dois pictogramas (que podem ser do vocabulário acessório ou do vocabulário essencial), de forma independente.
		4. O terapeuta deve utilizar o livro de comunicação ao direcionar sua fala à criança, apontando pictogramas da página de vocabulário essencial e do vocabulário acessório, modelando a sua comunicação.
		5. O terapeuta pode modelar, demonstrando à criança a formação de frases que deseja realizar.
		6. Quando a criança conseguir solicitar até 2 itens de abas diversas, folhear as abas de forma independente, apontar os pictogramas da frase EU + QUERO + dois pictogramas (que podem ser do vocabulário acessório ou do vocabulário essencial), de forma independente, pode seguir para promover o desenvolvimento da habilidade seguinte.
		7. O terapeuta deve orientar o cuidador a promover a aquisição da habilidade, devendo ser ensinado por meio de demonstração do terapeuta junto à criança e, em seguida, solicitar que o cuidador pratique junto à criança dentro do setting terapêutico. O terapeuta deve auxiliar o cuidador, dando feedbacks durante a prática, empoderá-lo quanto ao uso contínuo, bem como capacitar outros familiares e profissionais de contextos diversos.
		As novas palavras do vocabulário essencial e do vocabulário acessório estimuladas durante a sessão, devem ser estimuladas a serem mais utilizadas pela família.
**Ampliação morfossintática, lexical e das funções comunicativas**	A criança é capaz de formar frases com três ou mais palavras, com objetivos distintos:	1. O terapeuta pode inserir novas abas do vocabulário acessório como: sentimentos, noção de tempo, outros verbos, cumprimentos sociais, além das abas utilizadas na habilidade anterior.
	Desenvolvimento das funções comunicativas:	2. A atividade deve ser planejada de modo que promova o desenvolvimento das funções comunicativas com quatro ou mais pictogramas.
	● Função de informação/função interrogativa, com uso de perguntas com os pronomes interrogativos (quem, quando, qual, onde etc);	3. A criança, ao desejar realizar alguma das funções comunicativas e tenha dificuldade em encontrar o pictograma desejado, ou em folhear as abas, pode ser auxiliada com dica física, visual e/ou verbal, ou com modelagem, para apontar os quatro pictogramas de forma independente.
	● Função de comentários: realizar comentários, informações espontâneas, demonstrar algo, demonstrar dor, dar opinião, expressar ideias;	4. O terapeuta deve utilizar o livro de comunicação ao direcionar sua fala à criança, apontando pictogramas da página de vocabulário principal e do vocabulário acessório, modelando a sua comunicação.
	● Função de expressar sentimentos, gratidão;	5. O terapeuta pode modelar, demonstrando à criança a formação de frases que deseja realizar.
	● Função sociointerativa: realizar saudações, despedidas, agradecimentos, pedir desculpas e se exibir.	6. O terapeuta pode utilizar recursos visuais estruturados para promover o desenvolvimento da habilidade.
	A construção da frase ocorre de forma sequenciada, apontando para os pictogramas, podendo ser acompanhada da fala. Terá que utilizar com interlocutores e em contextos diversos para iniciar a aquisição da habilidade seguinte.	7. Quando a criança conseguir formar frases com quatro ou mais palavras, com objetivos pragmáticos distintos, utilizando itens de abas diversas, folheando as abas de forma independente, então pode seguir para promover o desenvolvimento da habilidade seguinte.
		8. O terapeuta deve orientar o cuidador a promover a aquisição da habilidade, devendo ser ensinado por meio de demonstração do terapeuta junto à criança e, em seguida, solicitar que o cuidador pratique junto à criança dentro do setting terapêutico. O terapeuta deve auxiliar o cuidador, dando feedbacks durante a prática, empoderá-lo quanto ao uso contínuo, bem como capacitar outros familiares e profissionais de contextos diversos
		9. As novas palavras do vocabulário essencial e do vocabulário acessório estimuladas para a formação de frases pela criança durante a sessão, devem ser estimuladas a serem mais utilizadas pela família.
**Diálogo**	A criança é capaz de apresentar as seguintes funções comunicativas:	1. O terapeuta pode inserir novas abas do vocabulário acessório, de acordo com a demanda da criança, da família, dos diversos contextos sociais e escolar da criança
	Função de relato: contar um fato ou recontar estórias;	2. A atividade deve ser livre de modo que promova o diálogo com uso das diversas funções comunicativas, manutenção do diálogo, criar, contar e recontar estórias, relatar fatos.
	Função imaginativa: criar uma estória ou contar piadas;	3. A criança, ao desejar realizar alguma das funções comunicativas e apresentar dificuldade em encontrar o pictograma desejado, pode ser auxiliada com reconstrução da frase com modelagem.
	Função conversacional: sustentar uma conversa;	4. O terapeuta deve utilizar o livro de comunicação ao direcionar sua fala à criança, modelando a sua comunicação.
	A construção da frase ocorre de forma sequenciada, apontando para os pictogramas, podendo ser acompanhada da fala. Terá que utilizar com interlocutores e em contextos diversos.	5. O terapeuta também pode modelar, demonstrando à criança a formação de frases que deseja realizar.
		6. O terapeuta pode utilizar recursos visuais estruturados para promover o desenvolvimento da habilidade.
		8. O terapeuta deve orientar o cuidador a promover a aquisição da habilidade, devendo ser ensinado por meio de demonstração do terapeuta junto à criança e, em seguida, inserir o cuidador na conversa, para que também utilize o livro de CAA junto à criança e ao terapeuta. O terapeuta deve auxiliar o cuidador, dando feedbacks durante a prática, empoderá-lo quanto ao uso contínuo, bem como capacitar outros familiares e profissionais de contextos diversos.
		9. A aquisição dessa habilidade demonstrará independência no uso do livro de comunicação DHACA, devendo ser iniciado o processo de alta assistida, em que o cuidador possa ter independência em inserir novas abas, caso seja necessário.

* Pictograma avulso – pictograma plastificado solto, avulso à prancha de comunicação fixa

## DISCUSSÃO

O método DHACA foi elaborado com base no conhecimento científico e na experiência clínica de fonoaudiólogas pesquisadoras que desenvolveram o método ao longo da trajetória profissional atuando na área de CAA com crianças autistas.

O método proposto utiliza um sistema robusto de comunicação que procura suprir as lacunas encontradas no que tange tanto à eficácia da CAA no desenvolvimento da comunicação, a viabilidade da implementação em contextos menos estruturados, mais naturalísticos, e a possibilidade de ser reproduzido clinicamente na intervenção fonoaudiológica dessa população^(5)^.

O processo de validação de um método de intervenção, particularmente na área da CAA no Brasil, constitui um fator inovador dada a ausência de métodos desenvolvidos para o contexto nacional.

O método tem como diferencial a base teórica sociopragmática e do desenvolvimento linguístico, com valorização do papel do parceiro de comunicação e uso em contextos diversos e naturais. É um método nacional que utiliza como base linguística o uso funcional da língua, desde a escolha do vocabulário ao uso das funções comunicativas. Além disso, considera as especificidades cultural e individual.

Pioneiro como método brasileiro de intervenção, descreve as habilidades a serem adquiridas, gradativamente e com base no desenvolvimento linguístico, por pessoas com TEA ao longo da intervenção, com objetivo final de adquirir a comunicação funcional. Utiliza um sistema robusto de comunicação, de fácil manuseio - num livro de comunicação com vocabulário essencial composto por pictogramas selecionados com base em um banco de palavras de alta frequência^(18)^ e vocabulário acessório.

Após a análise da concordância entre juízes, já na primeira avaliação, o resultado do IVC validou a aparência e conteúdo do método DHACA^(9)^. Entretanto destaca-se que, qualitativamente, foram apresentadas relevantes sugestões e os ajustes foram realizados, potencializando a aparência e conteúdo do método. Vale enfatizar que cinco juízes são profissionais com experiência clínica no uso de outros métodos de intervenção com CAA e validaram o método, o que fortalece a validação.

Dentre as sugestões destaca-se o preenchimento do fundo dos pictogramas do vocabulário essencial, na cor da classe gramatical correspondente, em vez de apenas a borda colorida. Essa mudança fortalece o conceito de sistema de codificação semântica de cores, que já era utilizado no método, mas apenas nas bordas, o qual agrupa conceitos em núcleos quanto ao seu papel gramatical, facilitando o uso, a memorização e auxiliando o desenvolvimento da gramática infantil.

Outra modificação foi a retirada dos pronomes demonstrativos “isto” e “aquilo”, e inclusão de “meu/minha”, “seu/sua.” Estudos apontam que a aquisição e uso de pronomes pessoais e possessivos estão relacionados à habilidade do interlocutor se reconhecer na relação com o outro e compreender a sua perspectiva e a do outro, ou seja, ao desenvolvimento de habilidades de comunicação social^(20)^. Além desses pronomes possessivos também serem de alta frequência, este recurso estimulará o uso dos pronomes das primeira pessoa nas pessoas com TEA que têm dificuldade quanto a esse aspecto^(21)^.

A seleção das palavras essenciais é fundamental na obtenção de bons resultados. Tanto a seleção quanto a organização do vocabulário são tarefas essenciais para o uso bem-sucedido da CAA, que devem fornecer acesso a um vocabulário grande, adequado ao desenvolvimento da comunicação e organizado para facilitar a recuperação de pictogramas^(18)^.

Sobre a sugestão no conteúdo do princípio da participação do parceiro de comunicação, ressaltou-se a relevância deste no processo de desenvolvimento das habilidades e uso do recurso em múltiplos contextos e ambientes, o que coaduna com o propósito do método com abordagem sociopragmática^(6)^.

Quanto ao uso de dicas, acredita-se que o uso inicial da dica física, ou seja, o uso de suportes sensoriais incluindo dicas de toque, propriocepção amplificada, entradas sensoriais multimodais (uso de áudio combinado com modelo visual para dar suporte ao movimento independente), favorece o desenvolvimento psicomotor e a aquisição de novas habilidades^(22)^.

Quanto à inserção de diferentes funções comunicativas no texto do princípio de “comunicação funcional”, foram incluídas a descrição de funções comunicativas no princípio de “desenvolvimento linguístico”, classificando-as em iniciais, intermediárias e avançadas; além da modificação das terminologias das habilidades do DHACA. Os sistemas robustos de comunicação permitem explorar e desenvolver as diferentes funções comunicativas, bem como o progresso morfossintático, semântico e pragmático^(8)^. Além disso, a seleção adequada do vocabulário essencial e acessório também possibilita que a criança se expresse de acordo com diferentes funções comunicativas^(18)^.

E quanto ao termo diálogo, foi modificada a nomenclatura da última habilidade, sendo alterado de habilidade narrativa por habilidade diálogo. Considerando que o objetivo da última habilidade é ter uma comunicação funcional, na qual ocorre um diálogo e não apenas a função comunicativa narrativa.

Diante das lacunas constatadas, quanto aos métodos de intervenção descritos cientificamente na área da CAA, enfatiza-se a importância da validação do conteúdo e aparência do método DHACA. Para além disso, a prática baseada em evidências na tomada de decisões é fundamental para elevar a qualidade da intervenção terapêutica.

O método DHACA abre caminho para outros estudos no âmbito da intervenção com o uso de CAA em pessoas com TEA, contribuindo para melhoria da prática clínica nos transtornos de linguagem associados ao TEA. Como perspectivas futuras, estão previstos estudos com o objetivo de avançar em outras etapas de validação com outras propriedades psicométricas.

## CONCLUSÃO

O estudo permitiu a validação de conteúdo e aparência do método DHACA, cumprindo com as etapas definidas na literatura para a validação.

Trata-se de um método inovador, que contribui para a fonoaudiologia brasileira, considerando a importância do uso de instrumentos validados e da prática baseada em evidências.

## Figures and Tables

**Figura 1A:** Página do Vocabulário essencial do Método DHACA

**Figura 2A:** Exemplo de abas dos vocabulários acessórios do Método DHACA
